# The α3 subunit of GABA_A_ receptors promotes formation of inhibitory synapses in the absence of collybistin

**DOI:** 10.1016/j.jbc.2021.100709

**Published:** 2021-04-24

**Authors:** Sven Wagner, ChoongKu Lee, Lucia Rojas, Christian G. Specht, JeongSeop Rhee, Nils Brose, Theofilos Papadopoulos

**Affiliations:** 1 Department of Molecular Neurobiology, Max Planck Institute of Experimental Medicine, Göttingen, Germany; 2 Diseases and Hormones of the Nervous System (DHNS), Inserm U1195, Université Paris-Saclay, Le Kremlin-Bicêtre, France; 3 Department of Molecular Biology, Universitätsmedizin Göttingen, Göttingen, Germany

**Keywords:** Gabra1, Gabra2, Gabra3, inhibitory synapse, glycine receptors, GlyR, ARHGEF9, hPEM-2, CA1, cornu ammonis 1, CB, collybistin, cDNA, complementary DNA, DG, dentate gyrus, DIV 4, *day in vitro* 4, E18, embryonic day 18, GABA, γ-aminobutyric acid, GABAergic, γ-aminobutyric acidergic, GABA_A_R, GABA type A receptor, GABA_A_R-α3, α3 subunit of GABA_A_R, GlyR, glycine receptor, mIPSC, miniature inhibitory postsynaptic current, NL, neuroligin, nRT, reticular thalamic nuclei, SR, stratum radiatum, TMD, transmembrane domain, VIAAT, vesicular inhibitory amino acid transporter

## Abstract

Signaling at nerve cell synapses is a key determinant of proper brain function, and synaptic defects—or synaptopathies—are at the basis of many neurological and psychiatric disorders. Collybistin (CB), a brain-specific guanine nucleotide exchange factor, is essential for the formation of γ-aminobutyric acidergic (GABAergic) postsynapses in defined regions of the mammalian forebrain, including the hippocampus and basolateral amygdala. This process depends on a direct interaction of CB with the scaffolding protein gephyrin, which leads to the redistribution of gephyrin into submembranous clusters at nascent inhibitory synapses. Strikingly, synaptic clustering of gephyrin and GABA_A_ type A receptors (GABA_A_Rs) in several brain regions, including the cerebral cortex and certain thalamic areas, is unperturbed in CB-deficient mice, indicating that the formation of a substantial subset of inhibitory postsynapses must be controlled by gephyrin-interacting proteins other than CB. Previous studies indicated that the α3 subunit of GABA_A_Rs (GABA_A_R-α3) binds directly and with high affinity to gephyrin. Here, we provide evidence (i) that a homooligomeric GABA_A_R-α3^A343W^ mutant induces the formation of submembranous gephyrin clusters independently of CB in COS-7 cells, (ii) that gephyrin clustering is unaltered in the neuronal subpopulations endogenously expressing the GABA_A_R-α3 in CB-deficient brains, and (iii) that exogenous expression of GABA_A_R-α3 partially rescues impaired gephyrin clustering in CB-deficient hippocampal neurons. Our results identify an important role of GABA_A_R-α3 in promoting gephyrin-mediated and CB-independent formation of inhibitory postsynapses.

Fast chemical synaptic transmission between neurons requires the orchestrated clustering of ionotropic neurotransmitter receptors in the postsynaptic plasma membrane. At γ-aminobutyric acidergic (GABAergic) postsynapses, inhibitory neurotransmission is mediated by γ-aminobutyric acid (GABA) acting through GABA type A receptors (GABA_A_Rs; for review, see (1)). These receptors are heteropentameric GABA-gated chloride channels that belong to the Cys-loop ligand-gated ion channel superfamily (2). They are encoded by 19 different genes, which are grouped into eight subclasses based on their sequence homologies (α1–6, β1–3, γ1–3, δ, ε, θ, π, and ρ1–3). Different subunit combinations of two α subunits, two β subunits, and a single γ subunit or δ subunit exist in distinct GABA_A_R subtypes (3). The subsets of GABA_A_Rs at synapses are typically composed of two α1 subunits, α2 subunits, or α3 subunits together with two β2 subunits or β3 subunits and a single γ2 subunit (4, 5). The γ2 is essential for the postsynaptic clustering of GABA_A_Rs (6).

Apart from the cognate GABA_A_Rs, core components of many GABAergic postsynapses are cell adhesion proteins, such as the neuroligins (NLs), particularly NL-2 and NL-4 (7, 8), the scaffolding protein gephyrin (9), and the guanine nucleotide exchange factor collybistin (CB, also known as ARHGEF9) (10), which together control GABA_A_R recruitment to synapses. The synaptic localization of gephyrin depends on the presence of certain GABA_A_R subtypes. For example, targeted deletion of the GABA_A_R-α1, GABA_A_R-α2, GABA_A_R-α3, or GABA_A_R-γ2 subunit results in a loss of synaptic gephyrin clusters (11, 12, 13, 14, 15, 16, 17). Vice versa, ablation of gephyrin expression by either antisense depletion in cultured neurons or by gene KO in mice prevents the clustering of glycine receptors (GlyRs) (9, 18) and α2- or γ2-containing GABA_A_Rs at developing postsynaptic sites (6, 19, 20). However, certain GABA_A_R subunits were previously found at synapses despite the absence of gephyrin. In the spinal cord, synaptic GABA_A_R-α1 localization is unaltered upon gephyrin KO, whereas the levels of α2, α3, β2/3, and γ2 at synapses are significantly reduced in gephyrin KOs (21). Specific effects of gephyrin depletion on receptor subtypes containing the α2 subunits and γ2 subunits have also been described in cultured hippocampal neurons, whereas synaptic clustering of GABA_A_R-α1 is unaffected by gephyrin KO (22).

An essential component of many, but not all, GABAergic synapses is the gephyrin-interacting protein CB (10, 23), which is a brain-specific guanine nucleotide exchange factor for the small GTPases Cdc42 and TC10 (24, 25). Genetic deletion of CB in mice leads to a massive reduction of synaptic gephyrin and γ2 subunit–containing GABA_A_Rs in certain regions of the forebrain, including the hippocampus and basolateral amygdala (26, 27). This loss of inhibitory postsynaptic proteins is accompanied by a substantial decrease in hippocampal GABAergic neurotransmission and significant changes in hippocampal synaptic plasticity, leading to impaired spatial learning and increased anxiety levels (26). In contrast, postsynaptic clustering of gephyrin and GlyRs in the brainstem and spinal cord and glycinergic neurotransmission in these regions are not altered by CB KO (26). In line with the functional defects observed in CB KO mice, mutations in the human CB homolog, hPEM-2, cause intellectual disability, epilepsy, and autism spectrum disorder (28, 29, 30, 31, 32, 33, 34, 35, 36, 37, 38, 39, 40). Strikingly though, GABAergic synapses in the cerebral cortex, certain thalamic areas, and other brain regions are unaffected by CB KO (26). This indicates that a substantial subset of GABAergic postsynapses is formed independently of CB and that the formation of those postsynapses must be controlled by gephyrin-interacting proteins other than CB. The identification of corresponding CB-independent mechanisms involved in the formation of inhibitory synapses is essential for a comprehensive understanding of inhibitory synapses and circuits in the brain, the identification of pathological mechanisms that cause epileptiform activity, intellectual disability, or autism spectrum disorder in GABAergic synaptopathies, and for the development of appropriate therapeutic strategies.

In addition to CB, gephyrin binds directly and with high affinity to the β subunit of GlyRs (41, 42, 43, 44, 45). Previous studies showed that gephyrin and GlyRs colocalize not only at the postsynaptic plasma membrane but also at the intracellular transport vesicles (46), and that active microtubule-dependent motor protein complexes interact and comigrate with GlyR-fusion and gephyrin-fusion proteins through neurite processes (47, 48, 49). Gephyrin also binds directly to certain α subunits of GABA_A_Rs, albeit with lower affinities than to the GlyR-β subunit (50, 51, 52, 53, 54, 55). More recently, the α2 subunit of GABA_A_Rs was shown to bind directly to both, gephyrin and CB (53, 54, 55). However, these studies were based on *in vitro* analyses using recombinantly expressed and purified intracellular loops or small peptides of receptor-binding domains that cannot fully account for the complexity of interactions between multiple components *in cellula*. One of the main obstacles in analyzing the interactions of individual GABA_A_R-α subunits with gephyrin in heterologous cells is that all of them accumulate in intracellular compartments upon their overexpression and do not travel to the cell surface alone (56, 57). Their cell surface expression requires their coassembly with β subunits and γ subunits in the endoplasmic reticulum (56, 57). Gephyrin has been previously shown to bind not only to α but also to β subunits of GABA_A_Rs (58). Thus, it is difficult to study and characterize the significance of individual GABA_A_R subunits for the submembranous clustering of gephyrin in cells separately, since coexpression of α subunits, β subunits, and γ subunits and their cell-surface localization as heteropentamers is required. Interestingly, a previous study found that the substitution of a single amino acid residue in the transmembrane domain (TMD) of the GABA_A_R-α1 subunit is sufficient to bypass the stringent assembly rules, permitting access of homomeric GABA_A_R-α1 complexes to the cell surface without the need for subunit coassembly (59).

Based on these findings (59), we mutated the corresponding TMD residues to enable cell surface assembly of homomeric α1 subunits, α2 subunits, or α3 subunits of GABA_A_Rs and analyzed the interaction of individual α subunits with gephyrin *in cellula*. We made three major observations: (i) the α3 subunit of GABA_A_Rs induces clustering of gephyrin at the plasma membrane independently of CB or additional accessory proteins. (ii) In contrast to α2 subunit containing GABA_A_Rs, the subcellular colocalization and synaptic clustering of GABA_A_R-α3 and gephyrin is unchanged in CB KO mice, pointing to an important role of the α3 subunit in priming gephyrin-mediated and CB-independent formation of GABAergic synapses. (iii) Recombinant expression of the α3 subunit partially rescues the impaired clustering of gephyrin in cultured CB KO hippocampal neurons, indicating that enhancing GABA_A_R-α3 function might be a potential tool toward improving therapeutic strategies that target GABAergic synapse dysfunction caused by loss of or aberrant CB function.

## Results

### Cell surface expression of the GABA_A_R-α3 subunit induces CB-independent formation of GFP–gephyrin submembranous microclusters

**Figure 1:**
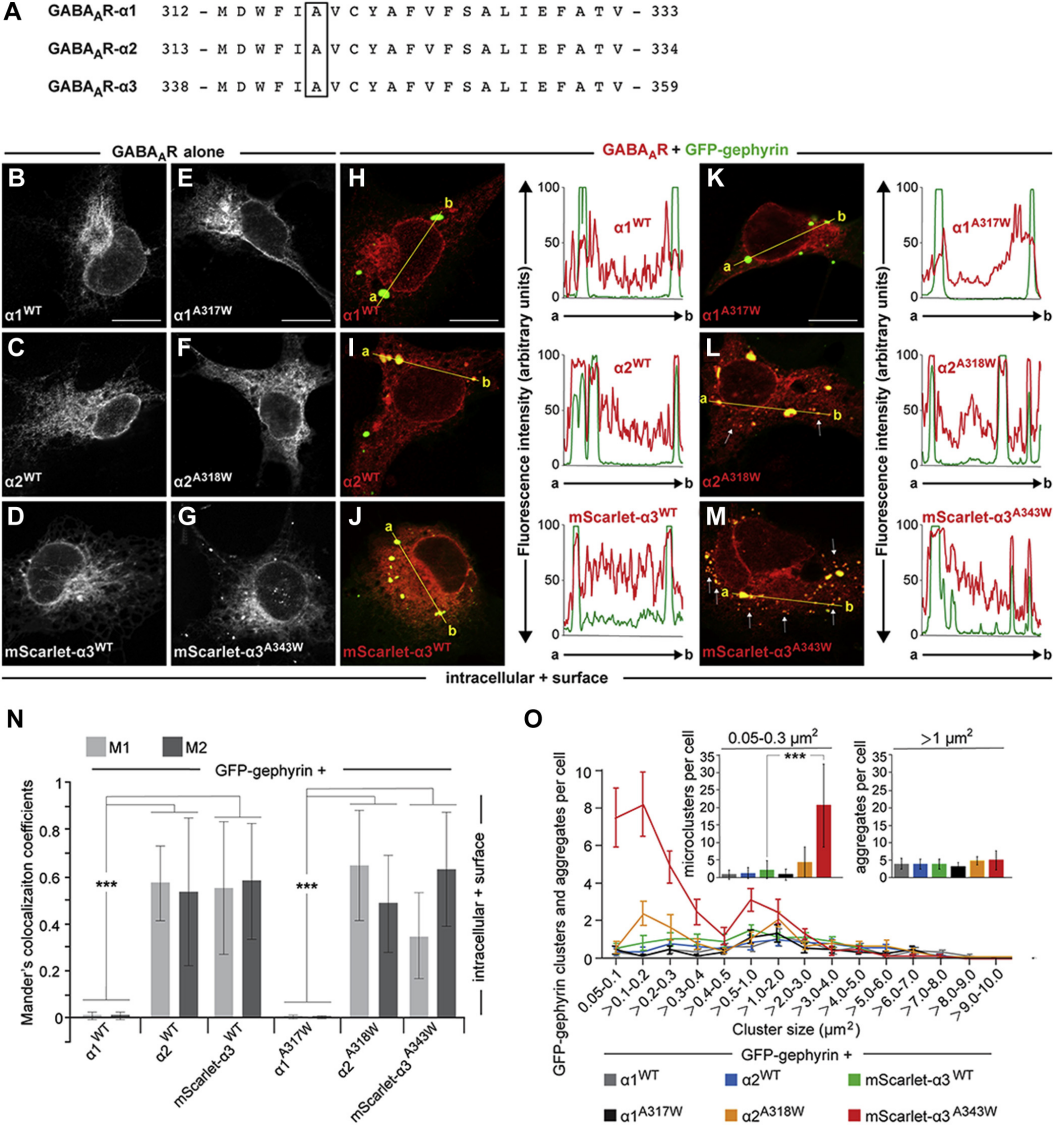
**Surface expression of the GABA**_**A**_**R-α3 subunit induces formation of GFP–gephyrin microclusters in COS-7 cells.***A*, alignment of a highly conserved primary sequence within the M3 domains of mouse α1, mouse α2, and rat α3 subunits of GABA_A_Rs used in this study. The boxed alanine (A) residue replaced by tryptophan (W) in α1 has been previously shown to induce surface expression of homomeric GABA_A_-α1 receptors (59). In the current study, the corresponding A residues of α1, α2, and α3 subunits were replaced by W, respectively. *B*–*G*, images of COS-7 cells expressing the WT α1, α2, and mScarlet-α3 subunits (*B*–*D*) or their corresponding A/W mutants (*E*–*G*). Total expression (intracellular + surface) of the different receptor subunits was visualized by cell permeabilization and immunostaining with subunit-specific antibodies (α1 and α2) or by the fused mScarlet fluorescent protein (α3). The scale bars represent 10 μm. *H*–*M*, images of COS-7 cells coexpressing GFP–gephyrin together with the WT α1, α2, or mScarlet-α3 (*H*–*J*) or their corresponding A/W mutants (*K*–*M*). *Right panels* indicate fluorescence intensity scans over the *yellow lines* in *H*–*J* and *K*–*M*, respectively (GFP–gephyrin, *green*; GABA_A_R subunits, *red*). Note that the highest peaks of α2 and mScarlet-α3 intensities, but not those of α1, colocalize with GFP–gephyrin. In addition, note that the mScarlet-α3 A343W mutant, and to a much lesser extent the α2 A318W mutant, induces the formation of GFP–gephyrin microclusters (indicated by *arrows* in *L* and *M*). The scale bars represent 10 μm. *N*, the degree of colocalization between the *red* (α subunit signal in permeabilized cells) and *green* (GFP–gephyrin) signals was statistically analyzed for the whole cells and expressed with the Mander's colocalization coefficients M1 and M2. M1 represents the fraction of the particular receptor subunit or its corresponding mutant, overlapping with GFP–gephyrin. M2 represents the fraction of GFP–gephyrin overlapping with the α subunits, as indicated. All calculations for the Mander's coefficients were performed by the ImageJ software, as described in *Experimental procedures* section. Data represent means ± SD of N = 10 cells per condition. ∗∗∗*p* < 0.001; unpaired two-tailed Student's *t* test. *O*, total numbers of GFP–gephyrin microclusters and aggregates from images of COS-7 cells coexpressing GFP–gephyrin together with α1 (WT or the A317W mutant), α2 (WT or the A318W mutant), or mScarlet-α3 (WT or the A343W mutant), as indicated. Counted puncta in N = 10 cells per condition were binned according to their size. *Insets*, relative fractions of microclusters (0.05–0.3 μm^2^; *left*) and aggregates (>1 μm^2^; *right*). Data in the insets represent means ± SD of N = 10 cells per condition. ∗∗∗*p* < 0.001; unpaired two-tailed Student's *t* test. GABA_A_R-α3, α3 subunit of GABA type A receptor.

Gephyrin was previously shown to interact with different affinities with the intracellular loops of GABA_A_ α1 subunit, α2 subunit, and α3 subunit (50, 51, 52, 53, 54, 55). Moreover, GABA_A_ α3 subunit displays the highest *in vitro* gephyrin affinity of all α subunits, with an estimated *K*_D_ of 5.3 μM for the full-length intracellular loop (53, 55). A previous study indicated that just a single substitution of a TMD alanine (A) residue with tryptophan (W) in the GABA_A_R-α1 subunit (Fig. 1*A*; substitution of the boxed A with W) permits access of homomeric α1 subunit GABA_A_Rs to the cell surface (59). Since this A residue is located within a highly conserved sequence in the M3 of the α1 subunit, α2 subunit, and α3 subunit (Fig. 1*A*), we performed the A/W substitution for all three subunits. This allowed us to study interactions of individual α subunits with gephyrin *in cellula*.

First, we expressed the individual WT α subunits and their corresponding A/W mutants in COS-7 cells in the absence of GFP–gephyrin (Fig. 1, *B*–*G*) and analyzed their subcellular distribution after fixation and cell permeabilization. Total (intracellular + surface) expressions were comparable between individual mutants and their corresponding WT proteins. Next, we coexpressed the individual α subunits or their corresponding A/W mutants together with GFP–gephyrin and analyzed their subcellular distribution and colocalization upon fixation and cell permeabilization (Fig. 1, *H*–*M*). GFP–gephyrin expressed alone (not shown) or together with WT α1 subunit, α2 subunit, or α3 subunit (Fig. 1, *H*–*J*) formed intracellular aggregates, as described previously (10, 41). In cells coexpressing GABA_A_R-α1^WT^, we consistently found no colocalization with intracellular GFP–gephyrin aggregates (Fig. 1*H*). Furthermore, analyses of fluorescence intensity plots (obtained by measuring the intensity profile along a straight line connecting GFP–gephyrin puncta; Fig. 1*H*; *right panel*) and Mander's coefficients for the whole cells (M1 and M2; Fig. 1*N*) indicated no correlation between the GFP–gephyrin aggregates and the α1^WT^ signals. In contrast, intracellular GFP–gephyrin aggregates were found to be colocalized with both, GABA_A_R-α2^WT^ and GABA_A_R-α3^WT^ (Fig. 1, *I* and *J*). Moreover, fluorescence intensity plots and Mander's coefficients indicated good correlation between GFP–gephyrin and α2^WT^ or α3^WT^ signals, respectively (Fig. 1, *I* and *J*; *right panels*; Fig. 1*N*). In cells expressing the α1^A317W^ mutant (Fig. 1*K*), no colocalization with GFP–gephyrin aggregates was observed, which is in line with the results obtained with α1^WT^ (Fig. 1*N*). Similar to α2^WT^ and α3^WT^, the α2^A318W^ (Fig. 1*L*) and α3^A343W^ (Fig. 1*M*) mutants colocalized with intracellular GFP–gephyrin aggregates, and corresponding fluorescence intensity plots indicated a clear overlap between the highest intensity peaks in both channels (Fig. 1, *L* and *M*; *right panels*). In addition, Mander's coefficients for the whole cells indicated good correlations between GFP–gephyrin and α2^A318W^ or α3^A343W^ signals, respectively (Fig. 1*N*).

More importantly, expression of α3^A343W^, and to a much lesser extent of the α2^A318W^ mutant, led to the formation of numerous smaller GFP–gephyrin clusters (so-called microclusters; indicated by *arrows* in Fig. 1, *L* and *M*). Accordingly, the mean total number of GFP–gephyrin microclusters (0.05–0.3 μm^2^) was significantly increased in COS-7 cells coexpressing GABA_A_R-α3^A343W^ (20.6 ± 11.9; Fig. 1*O*), as compared with cells expressing GABA_A_R-α3^WT^ (2.3 ± 2.6). In contrast, changes in the numbers of GFP–gephyrin microclusters in cells coexpressing GABA_A_R-α1^A317W^ (0.9 ± 1.6) or GABA_A_R-α2^A318W^ (4.4 ± 4.4) did not reach statistical significance, as compared with cells coexpressing GABA_A_R-α1^WT^ (0.9 ± 1.28) or GABA_A_R-α2^WT^ (1.3 ± 1.56), respectively (Fig. 1*O*).

Previous studies showed that GFP–gephyrin intracellular aggregates can be disassembled, so that gephyrin is recruited to membrane-associated microclusters, if a constitutively active splice variant of CB lacking the N-terminal SH3 domain, or if intrinsically inactive CB variants containing the N-terminal SH3 domain along with CB activators, such as NL-2, the α2 subunit of GABA_A_Rs, or the small Rho-like GTPase TC10, are coexpressed (7, 10, 25, 60, 61). However, in all previous studies, the presence of CB for redistributing gephyrin into microclusters in heterologous cells was obligatory. In contrast, our findings here indicate that mutations enabling cell surface expression of the GABA_A_R-α3 or GABA_A_R-α2 subunit induce gephyrin microcluster formation in the absence of CB.

**Figure 2:**
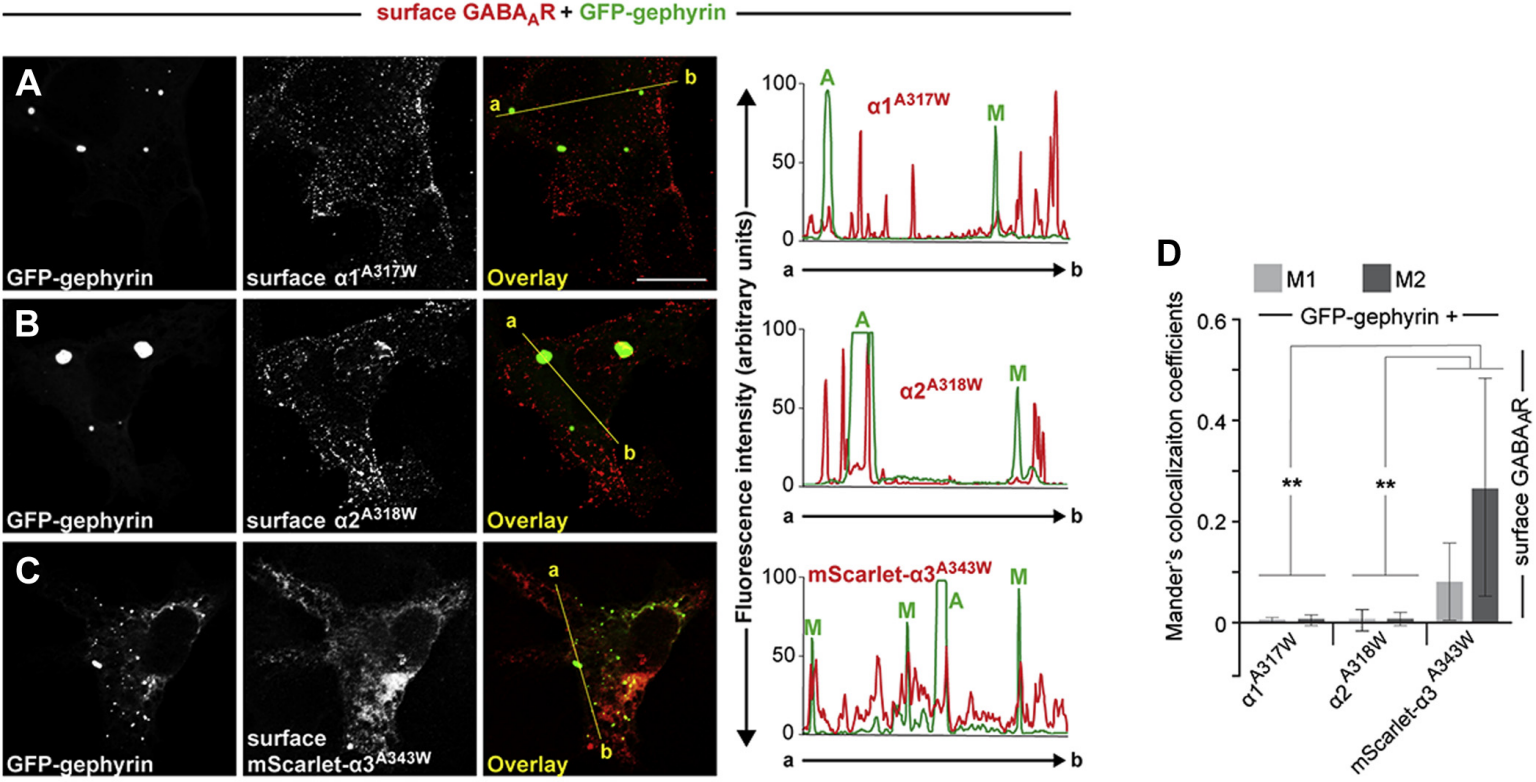
**The GABA**_**A**_**R-α3**^**A343W**^**mutant partially redistributes GFP–gephyrin into submembranous microclusters in COS-7 cells.***A*–*C*, images of COS-7 cells coexpressing GFP–gephyrin together with α1 A317W, α2 A318W, or the mScarlet-α3 A343W mutant, as indicated. Surface expression of the different receptor subunits in unpermeabilized cells was visualized by subunit-specific antibodies (α1, α2, or α3) binding to extracellular epitopes. *Right panels* indicate fluorescence intensity scans over the *yellow lines* in *A*–*C*, respectively (GFP–gephyrin, *green*; GABA_A_R subunits, *red*). Note that the highest peaks of mScarlet-α3 intensities, but not that of α1 or α2, colocalize with GFP–gephyrin submembranous microclusters (peaks marked with M). In contrast, intracellular GFP–gephyrin aggregates (peaks marked with A) do not colocalize with the immunoreactivities of the α subunits in unpermeabilized cells. The scale bar represents 10 μm. *D*, the degree of colocalization between the *red* (α1^A317W^, α3^A318W^, or α3^A343W^ immunoreactivities in nonpermeabilized cells) and *green* (GFP–gephyrin) signals was statistically analyzed for the whole cells and expressed with the Mander's colocalization coefficients M1 and M2. M1 represents the fraction of the α subunit mutants overlapping with GFP–gephyrin. M2 represents the fraction of GFP–gephyrin overlapping with α1^A317W^, α3^A318W^, or α3^A343W^, as indicated. Data represent means ± SD of N = 10 cells per condition. ∗∗*p* < 0.01; unpaired two-tailed Student's *t* test. GABA_A_R-α3, α3 subunit of GABA type A receptor.

Hannan and Smart (59) showed that the A/W mutation enables homomeric GABA_A_R-α1 subunits to traffic to the cell surface. To unequivocally demonstrate that the corresponding A/W mutations in the α2 and α3 subunits display a similar cell surface expression as α1, we again coexpressed the A/W mutants of the α1, α2, or α3 subunits together with GFP–gephyrin and analyzed their immunoreactivities upon fixation in nonpermeabilized cells using subunit-specific antibodies against extracellular epitopes. This revealed that the A/W mutations enabled all three α subunits to reach the cell surface (Fig. 2, *A*–*C*). As expected for nonpermeabilized cells, no colocalization between intracellular GFP–gephyrin aggregates and the immunoreactivities of GABA_A_R-α subunits were observed (see *overlays* in Fig. 2, *A*–*C*). In addition, fluorescence intensity profiles along a straight line through the GFP–gephyrin puncta indicated no correlation between the intracellular GFP–gephyrin aggregates (marked with *A*) and surface-labeled α subunits (Fig. 2, *A*–*C*; *right panel*). In contrast, GABA_A_R-α3^A343W^ immunoreactivities, but not those of GABA_A_R-α1^A317W^ or GABA_A_R-α2^A318W^, were partially colocalized with submembranous GFP–gephyrin microclusters (marked with M in the fluorescence intensity scans of Fig. 2, *A*–*C*). In addition, analyses of Mander's coefficients for the whole cells (M1 and M2; Fig. 2*D*) indicated no correlation between the GFP–gephyrin fluorescence and the α1^A317W^ or the α2^A318W^ surface signal but partial and significantly increased colocalization of the GFP–gephyrin signal with surface-localized mScarlet-a3^A343W^ (Fig. 2*D*).

Together, the results shown in Figures 1 and 2 demonstrate that surface expression of homomeric GABA_A_R-α3, the α subunit with the highest binding affinity for gephyrin as compared with α1 or α2 (55), induces the formation of submembranous GFP–gephyrin microclusters. Thus, α3 is the first GABA_A_R subunit shown to be capable of inducing submembranous gephyrin clustering in the absence of CB.

### CB-dependent clustering of gephyrin in neurons endogenously expressing the GABA_A_R-α2 subunit

Previous studies indicated that the α2 subunit of GABA_A_Rs binds with high affinity to CB and with low affinity to gephyrin (53, 54, 55). In contrast, the α3 subunit of GABA_A_Rs binds to gephyrin with the highest affinity estimated for GABA_A_R subunits but does not interact with CB (53, 55). Whereas the immunoreactivity of the GABA_A_R-α1 subunit is the most abundant and ubiquitous across the brain, the immunoreactivities of GABA_A_R-α2 and GABA_A_R-α3 subunits have more restricted, although distinct, distributions, being expressed at high levels only in specific brain areas (62). For example, GABA_A_R-α2 is expressed throughout the hippocampus, but not in the reticular thalamic nuclei (nRT), whereas GABA_A_R-α3 is abundantly expressed in nRT, an area rich in GABAergic interneurons (63) but less so or not at all in the different hippocampal areas (62). The respective distributions of the α2 and α3 subunits also indicate that they occur mostly in distinct neuronal populations (62).

**Figure 3:**
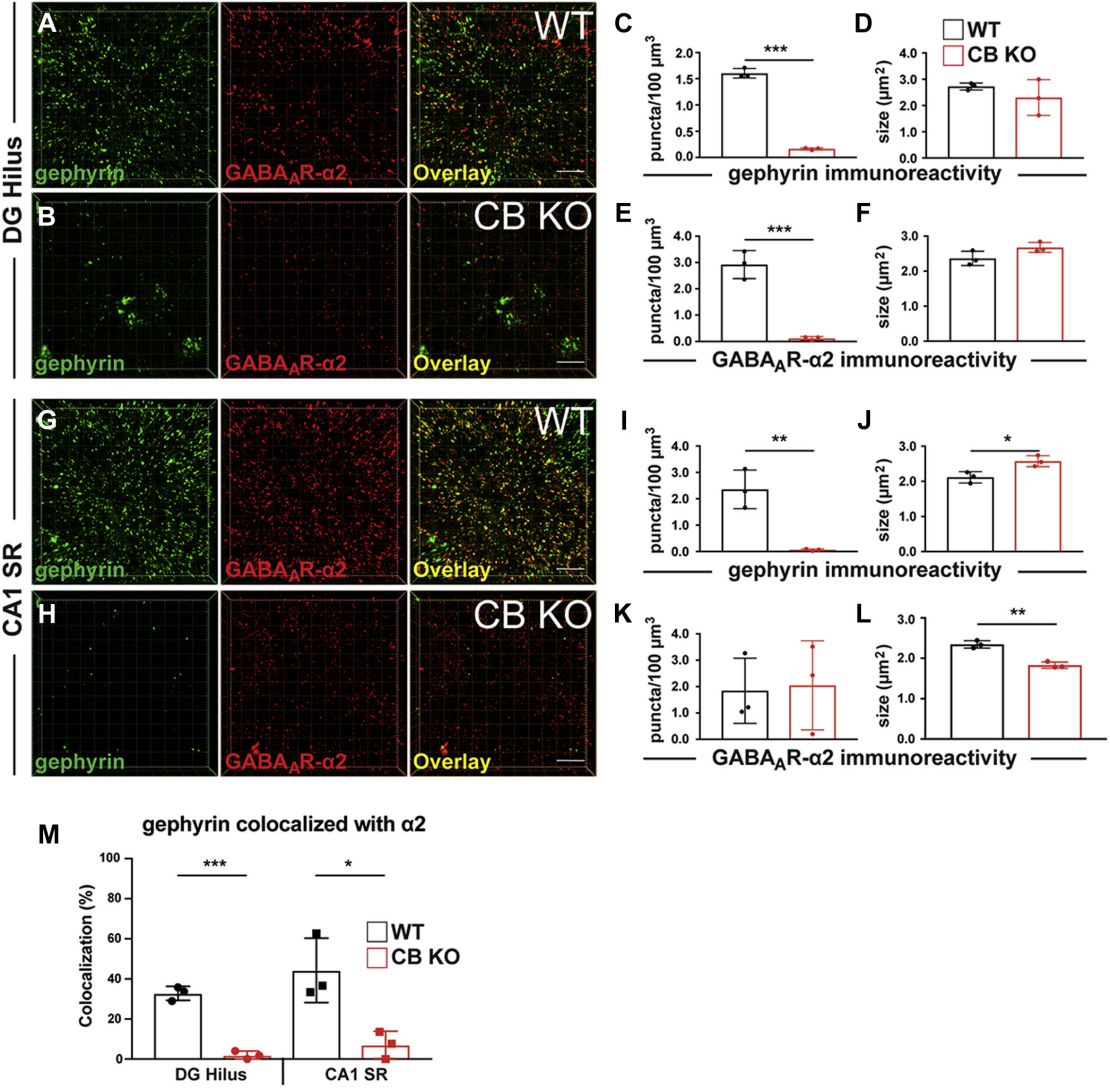
**The clustering of GABA**_**A**_**R-α2 in the hilus of the DG and CA1 SR of the hippocampus depends on both CB and gephyrin.***A* and *B*, representative snapshots of confocal 3D images of gephyrin/GABA_A_R-α2 colabeling in the hilus of the DG in slices derived from 8-week-old CB WT mice (*A*) or their CB KO littermates (*B*). Slices were processed for confocal microscopy, and images were generated using the Imaris software, as described in *Experimental procedures* section. In agreement with previous studies (26, 64), a strong reduction of both, gephyrin and α2 punctate immunoreactivities was observed in CB KO slices, as compared with controls. The scale bars represent 10 μm. Scale frames within the 3D images are composed of 5 × 5 μm squares. *C*–*F*, quantifications of gephyrin (*C* and *D*) and GABA_A_R-α2 (*E* and *F*) immunoreactive densities (*C* and *E*) and apparent sizes of puncta (*D* and *F*) in the hilus of 8-week-old CB WT mice (*black*) or their CB KO littermates (*red*). Each bar corresponds to mean values (±SD) obtained with sections from three individual brains per genotype. ∗∗∗*p* < 0.001; unpaired two-tailed Student's *t* test. *G* and *H*, representative 3D images of gephyrin/GABA_A_R-α2 costainings in the hippocampal CA1 SR in slices derived from 8-week-old CB WT mice (*G*) or their CB KO littermates (*H*). The scale bar represents 10 μm. *I* and *L*, quantifications of gephyrin (*I* and *J*) and GABA_A_R-α2 (*K* and *L*) immunoreactive puncta densities (*I* and *K*) and sizes (*J* and *K*) in the CA1 SR of 8-week-old CB WT mice (*black*) or their CB KO littermates (*red*). Note the strong reduction of gephyrin punctate immunoreactivity (*I*) and the reduction in the size of GABA_A_R-α2 puncta (*L*) in CB KO slices, as compared with controls. Each bar corresponds to mean values (±SD) obtained with sections from N = 3 individual brains per genotype. ∗*p* < 0.05, ∗∗*p* < 0.01; unpaired two-tailed Student's *t* test. *M*, quantifications of the percentages of gephyrin-immunoreactive puncta colocalized with GABA_A_R-α2 in the hilus of DG and CA1 SR of 8-week-old CB WT mice (*black*) or their CB KO littermates (*red*). Each bar corresponds to mean values (±SD) obtained with sections from N = 3 individual brains per genotype. ∗*p* < 0.05, ∗∗*p* < 0.01; unpaired two-tailed Student's *t* test. CA1, cornu ammonis 1;CB, collybistin; DG, dentate gyrus; GABA_A_R-α2, α2 subunit of GABA type A receptor; SR, stratum radiatum.

Previous studies indicated that CB deficiency leads to the loss of gephyrin clusters at a specific set of GABA_A_Rs and in distinct neuronal subpopulations of the mammalian forebrain (26, 27). However, in these studies, gephyrin and GABA_A_Rs were measured separately, disregarding the colocalization between gephyrin and specific GABA_A_R subunits in CB-deficient mice. As CB, in addition to its interaction with gephyrin (10), binds with high affinity to the GABA_A_R-α2 subunit, but not to the GABA_A_R-α3 subunit (55), we analyzed the densities and colocalization of gephyrin with α2 or α3 in different neuronal subpopulations with abundant to moderate expression of the individual subunits. For GABA_A_R-α2 subunit, we focused our analysis on two hippocampal regions with abundant expression of this subunit, the hilus of the dentate gyrus (DG) and stratum radiatum (SR) of the cornu ammonis 1 (CA1) hippocampal area (Fig. 3). In line with previous studies (26, 27, 64), the densities of gephyrin-immunoreactive puncta were significantly reduced in the brains of CB KO mice, as compared with their WT littermates (Fig. 3*C*; DG hilus: CB KO, 0.16 ± 0.02 puncta/100 μm^3^ *versus* WT, 1.60 ± 0.09 puncta/100 μm^3^; Fig. 3*I*; CA1 SR: CB KO, 0.06 ± 0.04 puncta/100 μm^3^ *versus* WT, 2.36 ± 0.73 puncta/100 μm^3^). The apparent sizes of the remaining gephyrin puncta in the DG hilus of CB KO animals were comparable to WT values (Fig. 3*D*; CB KO, 2.3 ± 0.68 μm^2^ *versus* WT, 2.72 ± 0.13 μm^2^). However, in the CA1 SR, the mean size of gephyrin puncta was significantly increased, as compared with controls (Fig. 3*J*; CB KO, 2.57 ± 0.16 μm^2^ *versus* WT, 2.11 ± 0.16 μm^2^). As previously shown (26), this is probably because of the accumulation of gephyrin in intracellular aggregates within the somata of isolated CA1 neurons. GABA_A_Rs containing the α2 subunit were previously shown to depend on both gephyrin and CB for postsynaptic localization (6, 19, 55). In line with this, the mean density of GABA_A_R-α2 immunoreactive puncta was significantly reduced in the hilus of CB KO mice as compared with controls (Fig. 3*E*; CB KO, 0.12 ± 0.07 puncta/100 μm^3^ *versus* WT, 2.92 ± 0.53 puncta/100 μm^3^). In contrast, in the CA1 SR, the GABA_A_R-α2 densities were similar between groups (Fig. 3*K*; CB KO, 2.05 ± 1.69 puncta/100 μm^3^ *versus* WT, 1.84 ± 1.24 puncta/100 μm^3^), even though the apparent sizes of the α2 subunit puncta were significantly reduced in the CA1 SR, as compared with WT (Fig. 3*L*; CB KO, 1.83 ± 0.08 μm^2^ *versus* WT, 2.34 ± 0.09 μm^2^). Moreover, the analysis of gephyrin-immunoreactive puncta colocalized with GABA_A_R-α2 puncta indicated a strong reduction in the fraction of colocalized puncta in the brains of CB KO animals, as compared with those of WT littermates (Fig. 3*M*).

The aforementioned data indicate that CB is indispensable for the clustering and/or stabilization of gephyrin and α2 subunit containing GABA_A_Rs, as well as for the colocalization of the two proteins at synapses.

### CB-independent clustering of gephyrin in neurons endogenously expressing the GABA_A_R-α3 subunit

**Figure 4:**
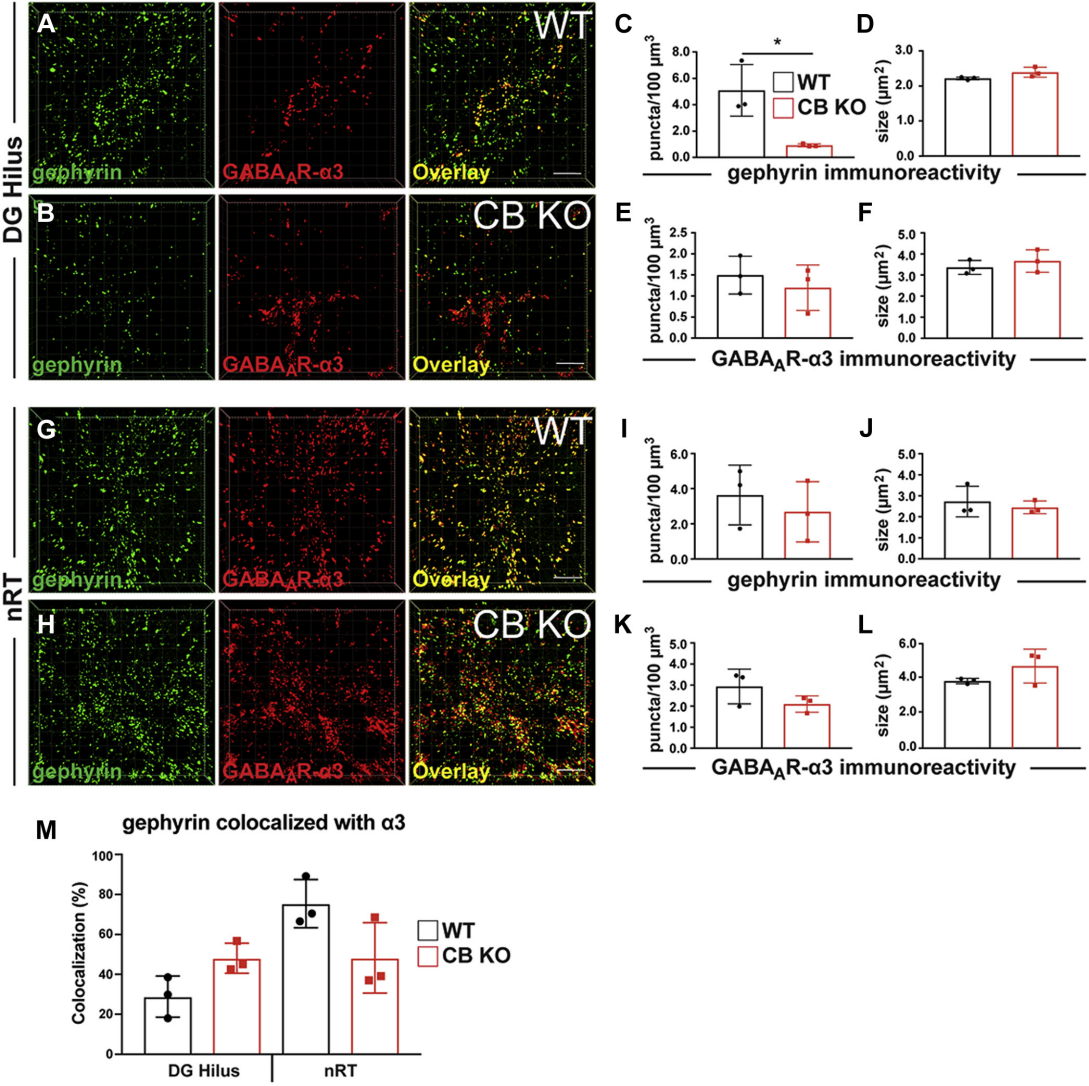
**CB-independent clustering of gephyrin and GABA**_**A**_**R-α3 in certain neuronal subpopulations with high endogenous GABA**_**A**_**R-α3 expression.***A* and *B*, representative confocal 3D images of gephyrin/GABA_A_R-α3 colabeling in the hilus of the DG in slices derived from 8-week-old CB WT mice (*A*) or their CB KO littermates (*B*). Note the strong reduction of gephyrin puncta in CB KO animals, as described previously (26). In contrast, the punctate immunoreactivity of GABA_A_R-α3 was similar between groups. In addition, note that a significant amount of the remaining gephyrin puncta in the hilus of the CB KO brains colocalize with GABA_A_R-α3 puncta. The scale bar represents 10 μm. Scale frames within the 3D images are composed of 5 × 5 μm squares. *C*–*F*, quantifications of gephyrin (*C* and *D*) and GABA_A_R-α3 (*E* and *F*) immunoreactive puncta densities (*C* and *E*) and sizes (*D* and *F*) in the hilus of 8-week-old CB WT mice (*black*) and their CB KO littermates (*red*). Each bar corresponds to mean values (±SD) obtained with sections from three individual brains per genotype. ∗*p* < 0.05; unpaired two-tailed Student's *t* test. *G* and *H*, representative confocal 3D images of gephyrin/GABA_A_R-α3 dual labeling in the nRT in slices derived from 8-week-old CB WT mice (*G*) or their CB KO littermates (*H*). The scale bar represents 10 μm. *I*–*L*, quantifications of gephyrin (*I* and *J*) and GABA_A_R-α3 (*K* and *L*) immunoreactive puncta densities (*I* and *K*) and sizes (*J* and *K*) in the nRT of 8-week-old CB WT mice (*black*) or their CB KO littermates (*red*). Note that both gephyrin (*I* and *J*) and GABA_A_R-α3 (*K* and *L*) values are similar between groups. Each bar corresponds to mean values (±SD) obtained with sections from N = 3 individual brains per genotype. *M*, quantifications of the percentages of gephyrin-immunoreactive puncta colocalized with GABA_A_R-α3 in the hilus of DG and nRT of 8-week-old CB WT mice (*black*) or their CB KO littermates (*red*). Note that colocalization percentages are not significantly different between CB KO animals (*red*) and their WT littermates (*black*). Each bar corresponds to mean values (±SD) obtained with sections from N = 3 individual brains per genotype. CB, collybistin; DG, dentate gyrus; GABA_A_R-α3, α3 subunit of GABA type A receptor; nRT, reticular thalamic nuclei.

To analyze the clustering of GABA_A_R-α3 in the absence of CB, we focused on two regions, the hilus of the DG, in which a small population of neurons were found to express high levels of α3, and the nRT, the region with the highest expression of GABA_A_R-α3 in the mouse brain (15, 16, 62). In contrast to GABA_A_R-α2, which is abundantly expressed in the hilus, the α3 immunoreactivity in this area is only sparse and restricted to few hilar neurons. Hilar areas encompassing neurons expressing the α3 subunit were selected in slices derived from CB KO and WT animals, and the densities of both gephyrin and α3 puncta were quantified. Again, we consistently found a strong reduction in the density of gephyrin puncta in the hilus of CB KO mice, as compared with WT controls (Fig. 4, *A*–*C*; CB KO, 0.92 ± 0.11 puncta/100 μm^3^ *versus* WT, 5.09 ± 1.96 puncta/100 μm^3^). In contrast, GABA_A_R-α3 puncta densities (Fig. 4*E*; CB KO, 1.19 ± 0.54 puncta/100 μm^3^ *versus* WT, 1.49 ± 0.45 puncta/100 μm^3^) and apparent sizes (Fig. 4*F*; CB KO, 3.67 ± 0.54 μm^2^ *versus* WT, 3.37 ± 0.33 μm^2^) in the hilus were comparable between groups. Furthermore, the percentages of gephyrin puncta colocalized with α3 puncta were not significantly different between groups (Fig. 4*M*, *left*). Interestingly, the percentage of gephyrin colocalized with α3 in the hilus of CB KO mice was markedly increased, as compared with WT, even though this effect did not reach significance (Fig. 4*M*, *left*; CB KO, 48.08 ± 7.56 % *versus* WT, 28.83 ± 10.29 %, *p* = 0.059). This result indicates that approximately half of the remaining gephyrin puncta in the hilus of CB KO animals are located at postsynapses of neurons expressing the GABA_A_R-α3 subunit (also compare *overlays* in Fig. 4, *A* and *B*).

In the nRT, the expression of GABA_A_Rs containing the α2 subunit was previously estimated to be very low to absent, whereas GABA_A_R-α3 is abundantly expressed (62). In this region, CB deficiency had no effect on the densities (Fig. 4, *I* and *K*; gephyrin CB KO, 2.69 ± 1.71 puncta/100 μm^3^ *versus* gephyrin WT, 3.64 ± 1.70 puncta/100 μm^3^; α3 CB KO, 2.1 ± 0.39 puncta/100 μm^3^ *versus* α3 WT, 2.94 ± 0.82 puncta/100 μm^3^) or on their apparent sizes (Fig. 4, *J* and *L*; gephyrin CB KO, 2.45 ± 0.30 μm^2^ *versus* gephyrin WT, 2.73 ± 0.73 μm^2^; α3 CB KO, 4.69 ± 0.97 μm^2^ *versus* α3 WT, 3.84 ± 0.15 μm^2^) of gephyrin and GABA_A_R-α3 immunoreactive puncta. In addition, colocalization analysis of gephyrin and α3 puncta revealed no significant differences between groups in the nRT (Fig. 4*M*, *right*).

These findings reveal no differences between CB KO brains and controls regarding the clustering of gephyrin and GABA_A_R-α3 in certain brain regions or neuronal subpopulations.

**Figure 5:**
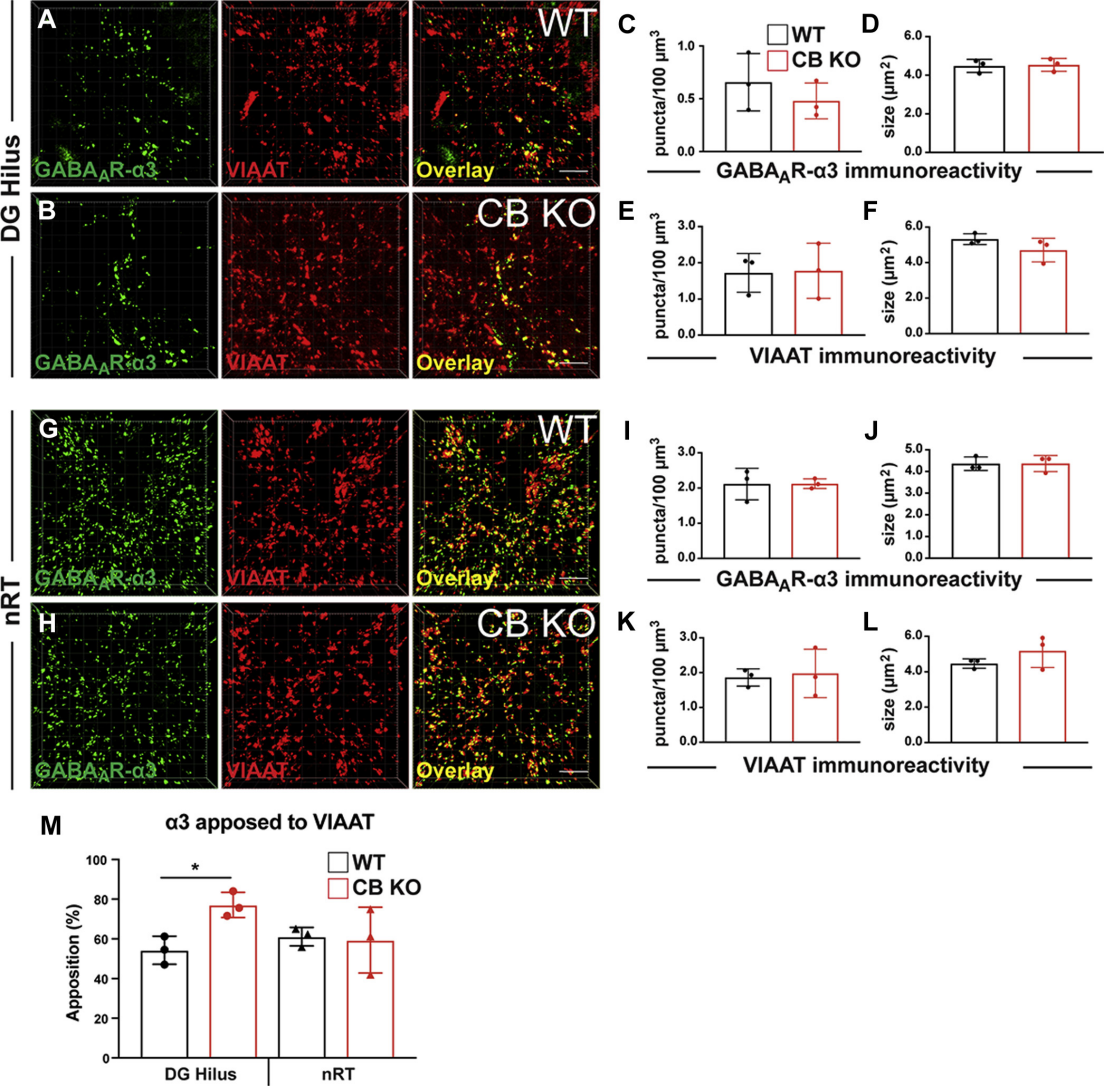
**CB-independent postsynaptic clustering of GABA**_**A**_**R-α3.***A* and *B*, representative confocal 3D images of GABA_A_R-α3/VIAAT costainings through the hilus of the DG in slices derived from 8-week-old CB WT mice (*A*) or their CB KO littermates (*B*). The scale bar represents 10 μm. *C*–*F*, quantification of the densities (*C* and *E*) and the apparent sizes (*D* and *F*) of GABA_A_R-α3 (*C* and *D*) and VIAAT (*E* and *F*) puncta in the hilus of 8-week-old WT (*black*) and CB KO mice (*red*). Note that both GABA_A_R-α3 (*C* and *D*) and VIAAT (*E* and *F*) values are similar between groups. *G* and *H*, GABA_A_R-α3/VIAAT labeling in the nRT in slices from 8-week-old CB WT mice (*G*) and their CB KO littermates (*H*). The scale bar represents 10 μm. Scale frames within the 3D images are composed of 5 × 5 μm squares. *I*–*L*, quantification of GABA_A_R-α3 (*I* and *J*) and VIAAT (*K* and *L*) immunoreactive puncta densities (*I* and *K*) and sizes (*J* and *K*) in the nRT of CB WT mice (*black*) or their CB KO littermates (*red*). Note that both GABA_A_R-α3 (*I* and *J*) and VIAAT (*K* and *L*) values are similar between groups (mean ± SD, slices from N = 3 brains per genotype). *M*, quantification of the fraction of GABA_A_R-α3 immunoreactive puncta apposed to VIAAT in the hilus of DG and nRT of 8-week-old mice. Note that the percentage of postsynaptic GABA_A_R-α3 puncta is significantly higher in the hilus of CB KO animals (*red*), as compared with the WT control group (*black*). Means ± SD from N = 3 brains per genotype. CB, collybistin; DG, dentate gyrus; GABA_A_R-α3, α3 subunit of GABA type A receptor; nRT, reticular thalamic nuclei; VIAAT, vesicular inhibitory amino acid transporter.

Furthermore, to particularly test whether CB deficiency affects the clustering of GABA_A_R-α3 at synaptic sites, we performed costainings for α3 and the vesicular inhibitory amino acid transporter (VIAAT), a well-characterized marker for inhibitory presynapses (65). Again, our quantifications in the hilus of the DG and nRT revealed no differences in the densities and sizes of both GABA_A_R-α3 and VIAAT-immunoreactive puncta between groups (Fig. 5, *A*–*L*). Furthermore, the percentages of postsynaptic GABA_A_R-α3 puncta in the nRT apposed to VIAAT were similar between groups (Fig. 5*M*, *right*). In contrast, the percentage of postsynaptic α3 apposed to VIAAT was significantly increased in the hilus of CB KO animals, as compared with their WT littermates (Fig. 5*M*, *left*; CB KO, 77.11 ± 6.33% *versus* WT, 54.26 ± 7.09%).

This result indicates that in the absence of GABA_A_Rs containing the α2 subunit in the hilus of CB KO mice, the synaptic clustering of the α3 subunit is increased.

### Partial rescue of gephyrin clustering in cultured CB KO hippocampal neurons exogenously expressing the GABA_A_R-α3 subunit

**Figure 6:**
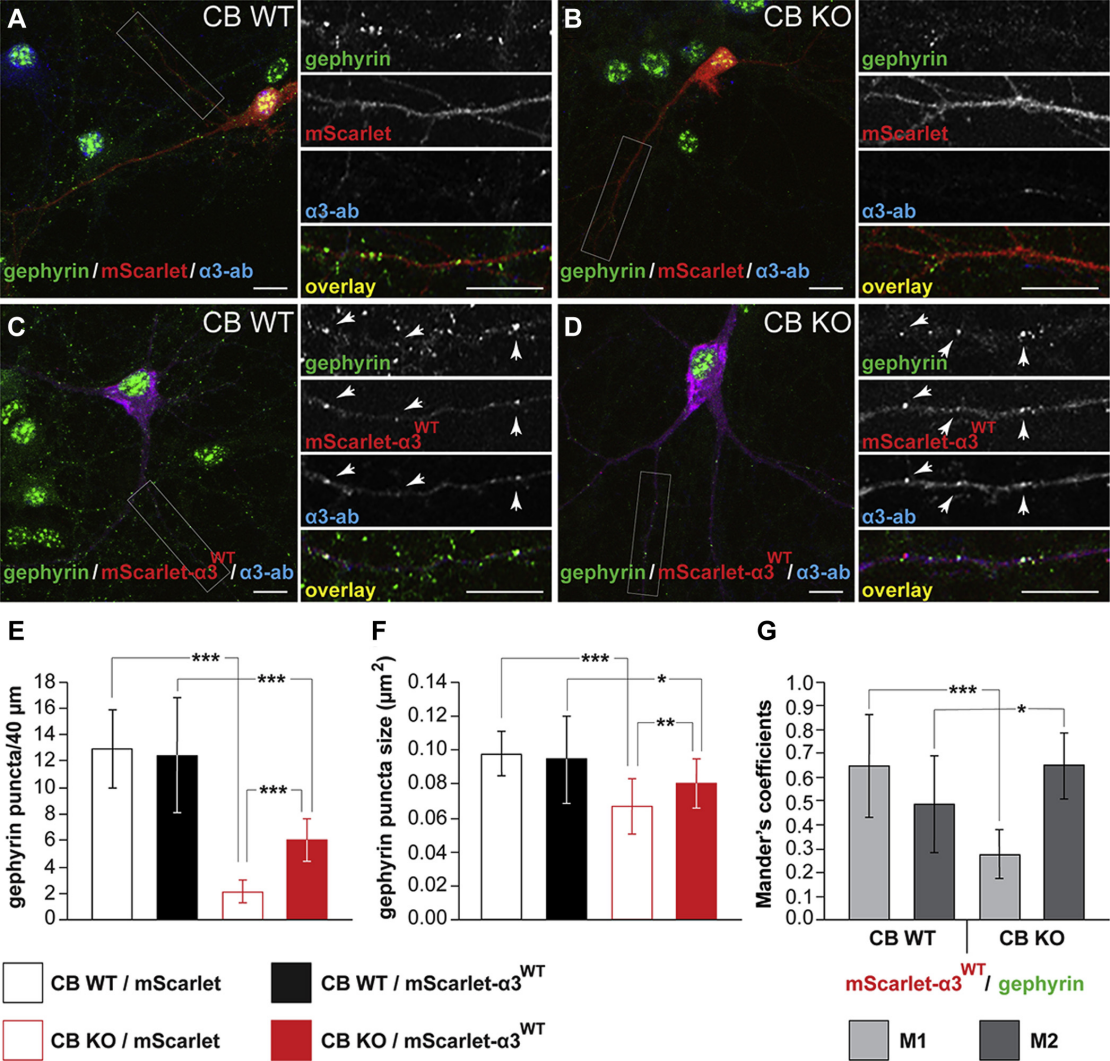
**Overexpression of mScarlet–GABA**_**A**_**R-α3 partially rescues the impaired gephyrin clustering in dissociated hippocampal neurons derived from CB KO mice.***A*–*C*, cultured hippocampal neurons from E18 CB KO mouse embryos (*B* and *D*) were transfected at DIV 4 with either mScarlet alone (*B*) or mScarlet–GABA_A_R-α3^WT^ (*D*). At DIV 14, the neurons were fixed and immunostained for gephyrin and GABA_A_R-α3. Cultured hippocampal neurons from E18 CB WT littermates, transfected with either mScarlet alone (*A*) or mScarlet–GABA_A_R-α3^WT^ (*C*), served as controls. *Right panels* show (from *top* to *bottom*) high-intensity magnifications of gephyrin immunoreactivities, mScarlet fluorescent signals, GABA_A_R-α3 immunoreactivities, and overlays of the boxed areas of *A*–*D*, respectively. *Arrowheads* indicate selected colocalized puncta in the three different channels. The scale bars represent 10 μm. *E* and *F*, quantifications of dendritic gephyrin-immunoreactive puncta densities (*E*) and sizes (*F*) in DIV 14 neurons from CB WT and CB KO animals, treated as indicated. In agreement with a previous study (26), gephyrin punctate staining was significantly reduced in the dendrites of CB KO neurons, as compared with controls. Note that overexpression of mScarlet–GABA_A_R-α3 partially rescues the impaired gephyrin clustering, as compared with CB KO neurons overexpressing mScarlet alone. Bars correspond to mean values (±SD) obtained from N = 10 individual neurons and n = 20 dendritic segments per condition and genotype. ∗*p* < 0.05, ∗∗*p* < 0.01, and ∗∗∗*p* < 0.001; unpaired two-tailed Student's *t* test. *G*, the degree of colocalization between the *red* (mScarlet-α3^WT^) and *green* (endogenous gephyrin) signals was statistically analyzed in the dendrites and expressed with the Mander's colocalization coefficients M1 and M2. M1 represents the fraction of mScarlet-α3^WT^ (*red*) overlapping with gephyrin (*green*). M2 represents the fraction of gephyrin overlapping with mScarlet-α3^WT^. All calculations for the Mander's coefficients were performed by the ImageJ software, as described in Experimental procedures section. Data represent means ± SD of 12 dendritic segments per condition. ∗*p* < 0.05, ∗∗∗*p* < 0.001; unpaired two-tailed Student's *t* test. CB, collybistin; DIV 4, *day in vitro* 4; E18, embryonic day 18; GABA_A_R-α3, α3 subunit of GABA type A receptor.

In the different areas of the rodent hippocampus, the expression of endogenous GABA_A_R-α3 is very low to absent (62), and previous studies on CB KO animals revealed the most severe defects in gephyrin and GABA_A_R clustering in these areas (26, 27). This indicates a possible compensation of CB loss by GABA_A_R-α3, in accord with our finding of CB-independent clustering of gephyrin in neuronal subpopulations that endogenously express the α3 subunit. We therefore studied the effects of recombinantly expressing GABA_A_R-α3 in cultured hippocampal CB KO neurons, which normally express very low levels of or no GABA_A_R-α3. We transfected cultured hippocampal neurons of CB KO embryos or their WT littermates on *day in vitro* 4 (DIV 4) with either mScarlet alone or with mScarlet–GABA_A_R-α3. At DIV 14, the neurons were fixed, permeabilized, and labeled with antibodies against GABA_A_R-α3 and endogenous gephyrin (Fig. 6, *A*–*D*). As previously described (62), the endogenous immunoreactivity of GABA_A_R-α3 in WT and CB KO neurons expressing mScarlet alone (high-intensity magnification panels of Fig. 6, *A* and *B*) was very low to absent. In agreement with a previous study (26), the densities of gephyrin puncta were significantly reduced in the dendrites of cultured hippocampal CB KO neurons expressing mScarlet alone, as compared with CB WT controls (Fig. 6, *A*, *B*, and *E*; CB KO/mScarlet, 2.15 ± 0.87 puncta/40 μm *versus* WT/mScarlet, 12.9 ± 2.97 puncta/40 μm). Similarly, the apparent sizes of gephyrin-immunoreactive puncta in CB KO neurons expressing mScarlet alone were reduced, as compared with controls (Fig. 6, *A*, *B*, and *F*; CB KO/mScarlet, 0.067 ± 0.016 μm^2^ *versus* WT/mScarlet, 0.098 ± 0.013 μm^2^). Overexpression of mScarlet–GABA_A_R-α3^WT^ in both CB KO neurons and WT controls led to the colocalization of the dendritic mScarlet-α3^WT^ signal with gephyrin and increased the level of GABA_A_R-α3 immunoreactivity (indicated by *arrowheads* in the zoomed panels of Fig. 6, *C* and *D*). Analysis of the Mander's coefficients M1 and M2 for selected dendritic segments showed good correlation of the mScarlet-α3^WT^ signal and the endogenous gephyrin signal in the dendrites of CB WT neurons (Fig. 6*G*; *left*). In CB KO neurons, we observed clear differences between M1 and M2 (Fig. 6*G*; *right*). The total mScarlet-α3^WT^ signal overlapping with the gephyrin signal (M1) was significantly reduced in CB KO neurons, as compared with the corresponding M1 values of CB WT cells (Fig. 6*G*; M1 of CB KO: 0.27 ± 0.1 *versus* M1 of CB WT: 0.65 ± 0.21). In contrast, the total dendritic gephyrin signal overlapping with the mScarlet-α3^WT^ signal (M2) was significantly increased in CB KO neurons, as compared with CB WT controls (Fig. 6*G*; M2 of CB KO: 0.65 ± 0.14 *versus* M2 of CB WT: 0.49 ± 0.2). In CB KO neurons expressing mScarlet–GABA_A_R-α3^WT^, densities and sizes of dendritic gephyrin puncta remained significantly reduced, as compared with WT/mScarlet-α3^WT^ controls (Fig. 6, *C*–*F*). Nonetheless, expression of mScarlet–GABA_A_R-α3^WT^ partially rescued the synaptic gephyrin immunoreactivity, as compared with CB KO neurons expressing mScarlet alone (Fig. 6, *B*, *D*, and *E*; mean density: 6.1 ± 1.62 puncta/40 μm; Fig. 6, *B*, *D*, and *F*; mean size: 0.08 ± 0.014 μm^2^).

These data indicate that the expression of exogenous GABA_A_R-α3 in cultured hippocampal CB KO neurons partially rescues the impaired clustering of gephyrin observed in CB KO cultures and significantly increases the colocalization of nascent gephyrin-immunoreactive puncta with mScarlet-α3^WT^ containing GABA_A_Rs, as compared with controls.

**Figure 7:**
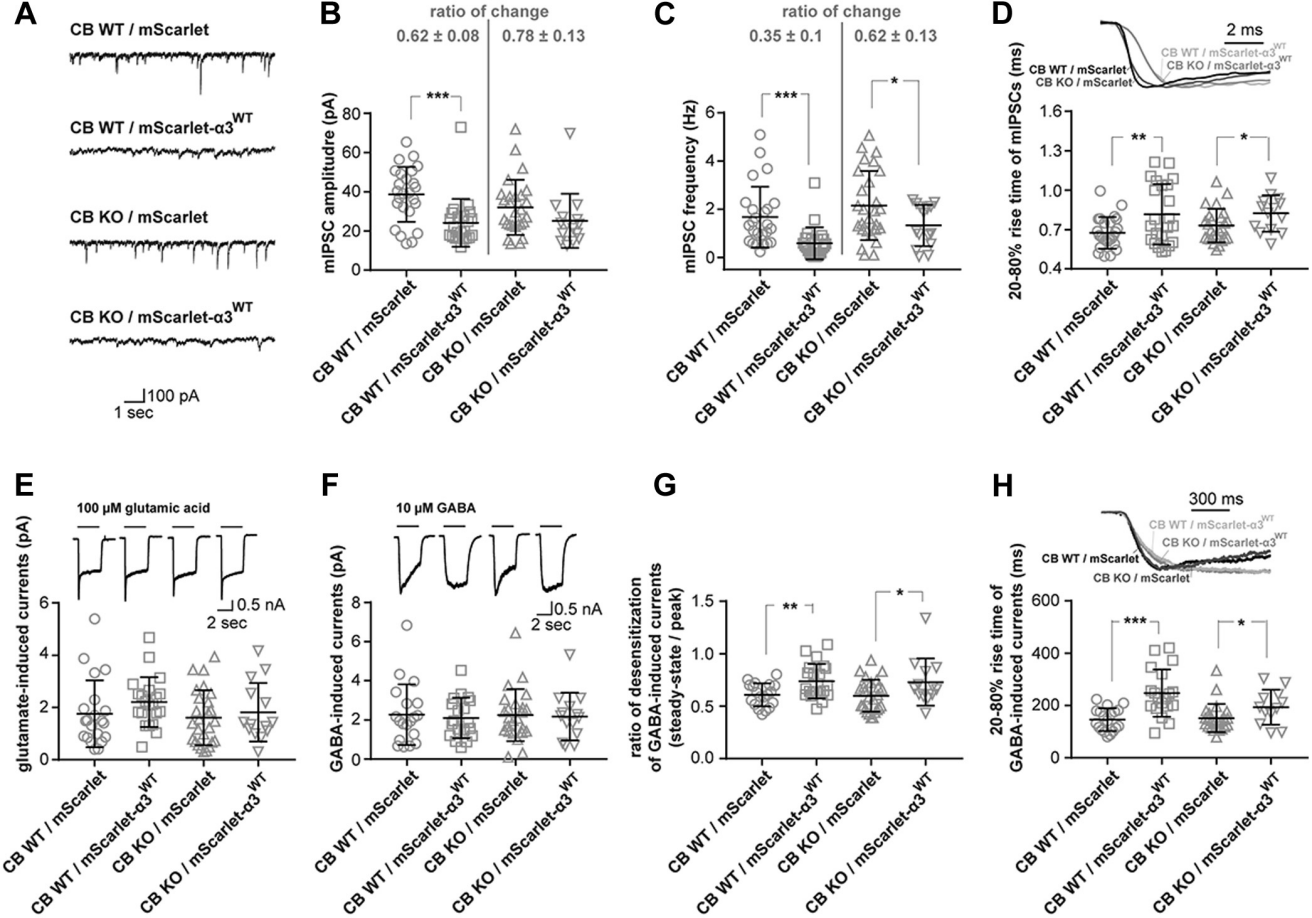
**Functional analysis of mScarlet–GABA**_**A**_**R-α3 expressing cultured hippocampal neurons.***A*, representative mIPSC traces recorded at a holding potential of −70 mV in the presence of 300 nM TTX from CB WT or CB KO cultured hippocampal neurons expressing either mScarlet alone or mScarlet-α3^WT^, as indicated. *B* and *C*, mean mIPSC amplitudes (*B*) and frequencies (*C*) in CB WT or CB KO neurons expressing mScarlet alone or mScarlet-α3^WT^, as indicated. Note that overexpression of mScarlet-α3^WT^ led to reduced mIPSC amplitudes and frequencies in both, WT and KO cells, compared with cells expressing mScarlet alone. Furthermore, note increased ratios of change in both mean amplitudes and frequencies of CB KO neurons, as compared with WT cells. *D*, representative mIPSC events (*top*), and mean values of times to peak (20–80% rise time) of mIPSC events, as indicated. *E*, representative events (*top*) and mean currents induced by exogenous application of 100 μM glutamate in cultured hippocampal neurons, as indicated. *F*, representative events (*top*) and mean currents induced by exogenous application of 10 μM GABA in cultured hippocampal neurons, as indicated. *G*, mean ratios of steady state/peak currents from GABA-induced responses recorded in CB WT or CB KO cultured hippocampal neurons expressing either mScarlet alone or mScarlet-α3^WT^, as indicated. *H*, representative events (*top*) and mean values of time to peak (20–80% rise time) of GABA-induced responses in cultured hippocampal neurons, as indicated. Data represent means ± SD of 14 to 27 cells per condition. ∗*p* < 0.05, ∗∗*p* < 0.01, and ∗∗∗*p* < 0.001; unpaired two-tailed Student's *t* test. Error estimates for ratios given in (*B*) and (*C*) were obtained by bootstrap analysis using 10,000 bootstrap resamples. CB, collybistin; GABA, γ-aminobutyric acid; GABA_A_R-α3, α3 subunit of GABA type A receptor; mIPSC, miniature inhibitory postsynaptic current; TTX, tetrodotoxin.

To determine the physiological consequences of the mScarlet-α3^WT^ overexpression in CB KO neurons, we recorded GABAergic miniature inhibitory postsynaptic currents (mIPSCs) in dissociated DIV 14–16 hippocampal neurons derived from CB KO embryos or their WT littermates, which were transfected at DIV 4 with either mScarlet alone or with mScarlet-α3^WT^. Interestingly, mean mIPSC amplitudes, frequencies, and rise times were not significantly different between CB WT and CB KO control neurons expressing mScarlet alone (Fig. 7, *A*–*D*; amplitudes: CB WT, 38.7 ± 14.04 pA *versus* CB KO, 32.08 ± 14.01 pA; frequencies: CB WT, 1.67 ± 1.26 Hz *versus* CB KO, 2.15 ± 1.43 Hz; rise times: CB WT, 0.67 ± 0.12 ms *versus* CB KO, 0.73 ± 0.13 ms). As previous recordings of GABAergic mIPSCs from CA1 pyramidal neurons in slices derived from CB KO mice and their WT littermates showed significantly reduced mean mIPSC amplitudes, frequencies, and rise times in CB KOs compared with controls (26), these results reveal qualitative differences in the composition of synaptic GABA_A_Rs in the dendrites of cultured hippocampal neurons, as compared with the *in vivo* situation. Accordingly, it was shown previously that the postnatal expression of GABA_A_R subunit mRNAs in the mammalian brain, including the hippocampus, exhibits a unique temporal and regional developmental profile *in vivo*, which may be altered in hippocampal cultures *in vitro* (66). In line with this, a previous study showed that in cultures of hippocampal gephyrin KO neurons, GABAergic transmission and expression of the gephyrin-independent α1 containing GABA_A_Rs were unchanged, as compared with WT controls (22). Thus, the high expression of α1-containing receptors in cultured neurons may mask the differences in dendritic inhibition between CB KOs and WT controls in our experiments.

Overexpression of mScarlet-α3^WT^ led to a reduction in the size of mIPSC events and slowed down their kinetics (see *exemplary traces* in Fig. 7*A*). In WT hippocampal neurons, mean mIPSC amplitudes (24.12 ± 12.19 pA) were significantly reduced compared with WT cells expressing mScarlet alone (Fig. 7*B*). Furthermore, mScarlet-α3^WT^–expressing neurons showed delayed events. In order to ensure that only real mIPSCs were recorded, events with a time-to-peak duration of more than 3 ms were excluded from our analysis. This type of analysis reported a significant reduction in the mean mIPSC frequencies of CB WT cells expressing mScarlet-α3^WT^ (0.59 ± 0.67 Hz) as compared with CB WT cells expressing mScarlet alone (Fig. 7, *A*–*C*).

Similarly, CB KO neurons expressing mScarlet-α3^WT^ showed significantly reduced mean mIPSC frequencies (1.33 ± 0.85 Hz). mIPSC amplitudes were also reduced (25.13 ± 13.84 pA), but this change did not reach statistical significance, as compared with CB KO cells expressing mScarlet alone (Fig. 7, *A*–*C*). In addition, mScarlet-α3^WT^–expressing neurons showed significantly increased mean rise times (CB WT, 0.82 ± 0.23 ms; CB KO, 0.83 ± 0.14 ms), as compared with neurons expressing mScarlet alone (Fig. 7*D*). Interestingly, a comparison of the ratios of change (mScarlet-α3^WT^/mScarlet) for both mean mIPSC amplitudes and mIPSC frequencies between CB WT and KO neurons yielded values closer to 1 in CB KO neurons, as compared with WT controls (Fig. 7*B*; ratios of change in mean amplitudes: CB WT, 0.62 ± 0.08 *versus* CB KO, 0.78 ± 0.13; Fig. 7*C*; ratios of change in mean frequencies: CB WT, 0.35 ± 0.1 *versus* CB KO, 0.62 ± 0.13). However, the indicated tendencies of higher ratios of change in CB KO cells for both, mIPSC amplitudes and mIPSC frequencies, were not statistically significant, compared with CB WT cells (*p* > 0.05 for both sets of data). Nevertheless, as synaptic localization of α3 subunit–containing GABA_A_Rs depends on the presence of a gephyrin scaffold at inhibitory postsynaptic sites (21), these tendencies of higher ratios of change in CB KO cells (Fig. 7, *B* and *C*) indicate that the relative amount of clustered gephyrin, capable of binding mScarlet-α3^WT^ at synapses, was partially increased in CB KO neurons expressing mScarlet-α3^WT^, as compared with CB KO neurons expressing mScarlet alone.

In order to ensure that the CB WT and KO neurons expressing mScarlet-α3^WT^ were healthy, as compared with neurons expressing mScarlet alone, we exogenously applied glutamic acid (100 μM) or GABA (10 μM) to activate all surface (synaptic + extrasynaptic) glutamate receptors and GABA_A_Rs (Fig. 7, *E* and *F*). No significant changes in the mean amplitudes of glutamate-induced (Fig. 7*E*) and GABA-induced (Fig. 7*F*) currents were apparent between the two genotypes and different groups (Fig. 7, *E* and *F*). Furthermore, the kinetics of glutamate-induced currents was similar between the two genotypes and experimental conditions (see *representative traces* in Fig. 7*E*). In contrast, the desensitization of GABA-induced currents was faster in cells of both genotypes expressing mScarlet, as compared with the cells expressing mScarlet-α3^WT^ (see *representative traces* in Fig. 7*F*). This indicates that the incorporation of mScarlet-α3^WT^ into functional pentameric GABA_A_Rs at synapses changed the kinetics of spontaneous desensitization of open channels. These results are in line with previous studies showing that the substitution of α3 for α1 in heteropentameric GABA_A_Rs reduces the apparent activating-site affinity and slows activation, desensitization, and deactivation of macroscopic currents (56, 67). Together, the unaltered kinetics of glutamate-induced currents and the mScarlet-α3^WT^–dependent changes in the kinetics of GABA-induced currents indicate that the reduced mean mIPSC amplitudes and frequencies, as well as the increased rise times observed in CB WT and CB KO neurons expressing mScarlet-α3^WT^ (Fig. 7, *A*–*D*), are due to the replacement of gephyrin-dependent α3 subunit for gephyrin-independent α1-containing GABA_A_Rs at inhibitory postsynaptic sites. In line with this, the ratios of desensitization (steady state/peak; Fig. 7*G*) and the rise times (Fig. 7*H*) of GABA-induced currents were significantly increased in CB WT and CB KO neurons expressing mScarlet-α3^WT^, as compared with neurons expressing mScarlet alone (Fig. 7, *G* and *H*). Together, the electrophysiological analyses of mIPSCs and GABA-induced currents indicated a significant incorporation of mScarlet-α3^WT^–containing GABA_A_Rs at synapses of both CB KO and CB WT neurons. Furthermore, the data shown in Figures 6 and 7 indicate that an increase in gephyrin clustering at synapses does not unconditionally correlate with an increase in GABA_A_R kinetics that are strongly dependent on their subunit composition. As α3 subunit–containing receptors depend on gephyrin for their postsynaptic clustering (21), however, our results indirectly indicate that overexpression of mScarlet-α3^WT^ is associated with an increase of gephyrin scaffolds at postsynaptic sites in the dendrites of CB KO neurons. Furthermore, the tendencies of higher ratios of change (mScarlet-α3^WT^/mScarlet) of the mean mIPSC amplitudes and frequencies in CB KO, compared with WT neurons, confirm our immunocytochemical data (Fig. 6) that the expression of α3 subunits in cultured hippocampal neurons leads to a partial rescue of the gephyrin clustering.

## Discussion

In the present study, we identified an important role of the α3 subunit of GABA_A_Rs in priming CB-independent and gephyrin-mediated formation of GABAergic synapses in specific regions of the mammalian forebrain. Evidence for a GABA_A_R-α3–dependent formation of certain inhibitory postsynapses in the absence of CB is provided by the following findings: (i) a homooligomeric GABA_A_R-α3^A343W^ mutant, which is trafficked to the cell surface, induces submembranous gephyrin clusters independently of CB in COS-7 cells. (ii) Gephyrin clustering is unaltered in those neuronal subpopulations of CB-deficient brains that endogenously express the GABA_A_R-α3. (iii) Exogenous expression of GABA_A_R-α3 partially rescues impaired gephyrin clustering in CB-deficient hippocampal neurons.

### Gephyrin clustering by GABA_A_R-α3

Homooligomeric mutant GABA_A_R-α3^A343W^ induces submembranous gephyrin clusters independently of CB in COS-7 cells. In all previous studies (7, 10, 25, 60, 61), the presence of CB was obligatory for the redistribution of gephyrin into such microclusters. In contrast, our findings clearly show that a mutation enabling cell surface expression of the GABA_A_R-α3 (59) induces formation of gephyrin microclusters in the absence of CB, and that these microclusters partially colocalized with GABA_A_R-a3 at the plasma membrane. Gephyrin binds directly to several GABA_A_R-α subunits, albeit with lower affinities than to the GlyR-β subunit (50, 51, 52, 53, 54, 55). More recently, the α2 subunit of GABA_A_Rs was shown to directly bind to both, gephyrin (with low affinity) and CB (with high affinity) (55), indicating that the α2 subunit is an essential determinant of CB activation and membrane anchoring at certain postsynapses (55, 60). In contrast, the GABA_A_R-α3 does not bind to CB but displays the highest *in vitro* gephyrin affinity of all α subunits with an estimated *K*_*D*_ of 5.3 μM for the full-length intracellular loop (53, 55).

In agreement with these studies, we consistently found that the α2 and α3 subunits, but not the α1 subunit, of GABA_A_Rs colocalized with intracellular GFP–gephyrin aggregates (>1 μm^2^) in COS-7 cells. Interestingly, both the β subunit of GlyRs and the α3 subunit of GABA_A_Rs strongly interact with gephyrin by occupying overlapping sites in the gephyrin E domain (54). Furthermore, a previous study indicated that glycinergic neurotransmission and clustering of GlyRs is unaffected in the spinal cord and brainstem of CB KO mice (26). Accordingly, the strong interaction of gephyrin with GlyRs and α3 subunit–containing GABA_A_Rs indicates that equivalent CB-independent mechanisms may modulate gephyrin-mediated clustering of GlyRs and α3-containing GABA_A_Rs at inhibitory synapses. In line with this, a recent study showed that gephyrin with its dimerizing E domain binds to the cytoplasmic loops of the GlyR-β subunit or the GABA_A_R-α3 subunit, and that the multivalent nature of GABA_A_Rs or GlyRs can polymerize gephyrin into a sheet-like assembly *via* phase separation (68). More importantly, this phase separation (and likely clustering at inhibitory synapses) of the gephyrin complexes with GlyRs or α3-containing GABA_A_Rs is regulated *via* phosphorylation of the gephyrin C domain (linker domain) or *via* binding of proteins, such as the dynein light chain, to the C domain of gephyrin (68).

### CB-independent gephyrin clustering in GABA_A_R-α3 expressing neurons

In agreement with previous data showing a high-affinity interaction between GABA_A_R-α2 and CB (55), the present study indicates that CB ablation causes significant defects in the clustering and colocalization of gephyrin and α2 subunit–containing GABA_A_R isoforms. However, areas or neuronal subpopulations with prominent expression of GABA_A_R-α3s, for example, the nRT, do not exhibit altered clustering and colocalization of gephyrin and GABA_A_R-α3. Given that GABA_A_R-α2 binds strongly to CB, but only with low affinity to gephyrin (55), a type of coincidence detection at GABAergic postsynapses as that previously proposed (69), where coinciding signals are amplified by a cooperative action of two different ligands at two different binding sites, might be significantly impaired in GABA_A_R-α2 expressing neurons of CB KO mice. In contrast, GABA_A_R-α3 binds strongly to gephyrin but not to CB (55). Thus, in the absence of CB, the interaction of GABA_A_R-α3 with additional, currently unknown, regulatory proteins in certain subpopulations of GABAergic postsynapses appears to be sufficient to retain postsynaptic clustering of gephyrin.

Different GABA_A_R subtypes display differential regional distributions (62). The most prevalent subunit combination is the triplet α1/β2,3/γ2, detected in numerous cell types throughout the brain, whereas other triplets such as the α2/β2,3/γ2 and α3/β2,3/γ2 were identified only in discrete cell populations (62). In the hilus of the DG, the α2 subunit of GABA_A_Rs dominates at inhibitory postsynaptic sites. Here, CB deficiency leads to a strong reduction of postsynaptic gephyrin and α2 cluster densities and sizes in these areas. The majority of the remaining gephyrin clusters in the hilus of CB KO animals colocalize with GABA_A_Rs in neurons expressing the α3 subunit. The sparse distribution of these GABA_A_R-α3 expressing neurons, with their dendritic trees restricted next to the border of the granule cell layer, indicates that they are highly differentiated groups of interneurons (70, 71). However, additional work will be required to specify the morphological and electrophysiological identity of the hilar interneurons that are insensitive to CB deficiency with regard to gephyrin and GABA_A_R-α3 postsynaptic clustering.

In nRT neurons, which selectively express the α3 subunit, none of the other five α subunits were detected in brain slices derived from α3-KO mice (15). Moreover, clustering of gephyrin was disrupted in these cells (15), providing additional evidence that in the neuronal subpopulation of the nRT, the GABA_A_R-α3, but not CB, constitutes one of the major determinants for gephyrin postsynaptic clustering. In the CA1 pyramidal neurons, which express both α1 and α2, but not the α3 subunit of GABA_A_Rs, α2 KO mice show significantly reduced gephyrin postsynaptic clustering but preserved perisomatic inhibition, which was proposed to be mediated by gephyrin-independent α1-containing GABA_A_Rs (17, 21, 72). In the current study, our immunolabeling analyses on slices derived from CB KO animals revealed qualitative differences in the clustering of α2-containing GABA_A_Rs in the CA1 SR that were not detected in previous studies (26), mainly because of the lack of high-resolution microscopy techniques and processing software. Whereas gephyrin immunoreactivity was strikingly reduced in the CA1 SR, our statistical analyses of the GABA_A_R-α2 immunoreactive puncta in 3D confocal images processed with the Imaris software (Bitplane) indicated significant differences in the apparent cluster sizes, but not in the densities of the α2 subunit in the CA1 SR of CB KO mice, as compared with controls. In contrast, in DG hilar neurons of CB KO mice, the strong reduction in gephyrin clustering was accompanied by an equivalent reduction in the clustering of GABA_A_R-α2. Together, these findings indicate that in particular hippocampal areas, such as the CA1 SR, the densities of GABA_A_R-α2 remain unaltered in the absence of both postsynaptic gephyrin and CB. The reason for the unchanged densities of α2 in CA1 pyramidal cells of CB KO mice is currently unknown but may reflect the activation of compensatory mechanisms and the involvement of scaffolding proteins other than gephyrin, such as dystrophin and its associated glycoprotein complex (17, 73, 74), in the clustering and stabilization of GABA_A_Rs at certain GABAergic postsynapses.

### GABA_A_R-α3 compensates the effects of CB loss on gephyrin clustering

In cultured hippocampal neurons prepared from embryonic day 18 (E18) mouse embryos, pyramidal neurons account for the vast majority of the total neuronal population, because in late-stage embryos, the generation of pyramidal cells is essentially complete, but the generation of dentate granule cells has not yet begun (75). Our study demonstrates CB-independent clustering of gephyrin in neuronal subpopulations that endogenously express GABA_A_R-α3. In contrast, in regions such as the CA1 SR, where α1 and α2, but not α3, are expressed, gephyrin-immunoreactive puncta are strikingly reduced in CB KO animals. This prompted us to study the effects of recombinantly expressing the GABA_A_R-α3 subunit in cultured pyramidal cells derived from the brains of CB KO embryos, as compared with WT controls.

Our functional analyses of control CB WT and CB KO neurons expressing mScarlet alone indicated no significant differences in mean mIPSC amplitudes, frequencies, and rise times between genotypes. This result is in line with a previous study showing that in cultured hippocampal neurons from gephyrin KO mice, GABAergic neurotransmission is unaffected, probably because of the high expression of GABA_A_Rs containing the gephyrin-independent α1 subunit (22). Overexpression of mScarlet–GABA_A_R-α3^WT^ in both CB WT and CB KO neurons led to a significant reduction in the size of the mIPSC events and slowed down their kinetics. This is probably because of the replacement of α1/β2,3/γ2 for mScarlet-α3/β2,3/γ2 receptors at inhibitory postsynaptic sites, revealing a dependence of the GABA_A_R gating kinetics on the α subunit isoform, as previously shown (67). As α3-containing GABA_A_Rs depend on gephyrin for their synaptic localization (21), the mScarlet-α3–induced changes in the GABA_A_R kinetics of CB KO neurons predicted the presence of a gephyrin scaffold at inhibitory postsynaptic sites.

Indeed, overexpression of mScarlet–GABA_A_R-α3 in CB KO neurons partially rescued the defects in dendritic gephyrin clustering. Both densities and sizes of gephyrin clusters were significantly increased in the dendrites of CB KO neurons expressing mScarlet-α3^WT^, compared with CB KO neurons expressing mScarlet alone. Moreover, the Mander's coefficients indicated that the increased fraction of gephyrin-immunoreactive puncta in the dendrites of CB KO neurons overlapped with the mScarlet-α3^WT^ signal. As mentioned previously, this result is in line with a recent study showing that the binding of GABA_A_R-α3 to the E domain of gephyrin induces clustering of gephyrin–GABA_A_R complexes at inhibitory synapses (68). However, densities and sizes of gephyrin clusters in CB KO cells expressing mScarlet-α3^WT^ remained significantly reduced, compared with CB WT neurons expressing mScarlet-α3^WT^, meaning that the rescue of gephyrin clustering in CB KO pyramidal neurons was partial. Accordingly, the ratios of change (mScarlet-α3^WT^/mScarlet) of both mean mIPSC amplitudes and mIPSC frequencies were less pronounced in CB KO neurons than in WT controls, suggesting that the fraction of clustered gephyrin was in fact partially increased upon overexpression of mScarlet-α3^WT^.

In view of the previously postulated function of the α subunits of GABA_A_Rs as prime mediators of specific targeting (76), different GABA_A_R-α subunits may bind directly or indirectly to different regulatory proteins. In the absence of CB, the interaction of GABA_A_R-α3 with gephyrin in hippocampal pyramidal neurons may require the involvement of other, currently unknown, regulatory proteins. The predominant expression of the α3 subunit of GABA_A_Rs in GABAergic interneurons, as opposed to glutamatergic neurons (62, 77), indicates a more important role of α3-containing GABA_A_Rs in localizing gephyrin at postsynaptic sites of interneuronal synapses. Thus, in glutamatergic pyramidal cells, the molecular machinery required for GABA_A_R-α3–mediated stabilization of gephyrin scaffolds at postsynaptic sites may be incomplete. Nevertheless, the partial rescue of the gephyrin clustering observed in cultured CB KO pyramidal neurons upon GABA_A_R-α3 overexpression indicates that manipulating expression of the α3 subunit in neuronal circuits affected in the absence of CB may be a potential target for developing novel therapeutic tools.

Loss of CB in mice results in increased levels of anxiety and impaired spatial learning (26). Accordingly, several mutations of the CB gene (ARHGEF9; Online Mendelian Inheritance in Man number: 300429) in patients have been implicated in epilepsy, X-linked intellectual disability, aggressive behavior, anxiety and, in one case, with hyperexplexia (29, 30, 31, 34, 35, 42, 78). Intriguingly, the anxiolytic activity of diazepam (79) is mediated primarily by GABA_A_Rs α2/β/γ2 (80) and under conditions of high receptor occupancy, also by GABA_A_Rs containing the α3 subunit (81, 82). One promising strategy in mouse models that has recently gained substantial attention is the use of viral vectors, particularly adeno-associated viral vectors, to introduce therapeutic tools in a circuit-specific manner (83). Thus, in cases in which directly targeting the mutated CB gene is unsuccessful, adeno-associated viral-mediated GABA_A_R-α3 expression in specific circuits affecting anxiety levels may be a promising new therapeutic strategy.

## Experimental procedures

### Animals

The generation and characterization of CB KO mice has been previously described (26). All mice used in the current study were housed in the Max Planck Institute of Experimental Medicine Animal Care Facility in Göttingen, and all procedures were approved by the Institutional Animal Care and Use Committee.

### cDNA constructs

The GFP–gephyrin plasmid has been described previously (84). The pcDNA3.1-gabra1 and pcDNA3.1-gabra2 constructs were kindly provided by Mr Yoshimitsu Sasa, Director of the Wako Administrative Division of the RIKEN Institute. The mScarlet_C1 vector and a FUGW-mScarlet-Gabra3, in which the GABA_A_R-α3 subunit was tagged with mScarlet after the signal peptide was generated by C. G. S. This construct contained an undesired point mutation (I342M). Upon cloning of the mScarlet-Gabra3 complementary DNA (cDNA) into the XbaI/EcoRI sites of the pKH3-vector (Addgene), correction of the undesired point mutation (M342 → I) in the pKH3-mScarlet-Gabra3 plasmid, as well as the introduction of the A317W, A318W, or A343W point mutations in the pcDNA3.1-gabra1, pcDNA3.1-gabra2, or the pKH3-mScarlet-Gabra3 plasmid, respectively, were performed by site-directed mutagenesis using the QuikChange protocol (Stratagene). Sequencing of the full open reading frames was performed in each case to validate constructs.

### Antibodies

The following primary antibodies were used for immunohistochemistry and immunocytochemistry: monoclonal mouse antigephyrin (mAb7a; Connex; 1:2000); polyclonal rabbit anti-GABAAR-α1 (AB5592; Chemicon; 1:1000), polyclonal rabbit anti-GABAAR-α2 (224103; Synaptic Systems; 1:1000), polyclonal rabbit anti-GABAAR-α3 (224303; Synaptic Systems; 1:1000), and polyclonal guinea pig anti-VIAAT (131004; Synaptic Systems; 1:1000). The following secondary antibodies were used for immunohistochemistry and immunocytochemistry: Alexa Fluor 488, 555, or 633, goat antimouse, goat anti-rabbit or goat antiguinea pig (Invitrogen; 1:2000). For nuclear stainings of brain slices, 4′,6-diamidino-2-phenylindole dihydrochloride (D1306; ThermoFisher Scientific) was used.

### Transfections and immunocytochemistry of COS-7 cells

COS-7 cells (CRL-1651) were purchased from American Type Culture Collection (LGC Standards GmbH). The cells were tested independently and certified to be free of *mycoplasma*. Transfections and immunocytochemistry were performed as described previously (36, 61). The transfection parameters were optimized in order to achieve low and comparable expression levels of all recombinant proteins analyzed. COS-7 cells were plated in 24-well plates on 12-mm coverslips, and 200 ng of each cDNA were used per well. The empty pcDNA 3.1 vector was used to equalize the total amount of DNA per transfection to 400 ng. Cells were transfected with Lipofectamine 2000 (Invitrogen) following the manufacturer's protocol, fixed 16 h after transfection, and stained, as described previously (25). Images were collected either with an Axio-Imager Z1 equipped with a Zeiss apochromat 63× objective and an Apotome module (Zeiss) or an inverse Leica DMIRE2 microscope equipped with a 63× oil-immersion objective and connected to a Leica TCS SP2 AOBS confocal laser scanning setup (Leica Microsystems). ImageJ (https://imagej.nih.gov/ij/) was used to analyze immunolabeling from images processed with standardized intensity thresholding. Briefly, the images were opened with ImageJ and, upon splitting the channels, the same intensity thresholds on the different GFP–gephyrin and GABA_A_R channels were constantly applied. Subsequently, the images were converted to a mask, single cells were selected using the “freehand selection” tool, and particle numbers and sizes between 0.05 and 20 μm^2^ were automatically analyzed by using the “Analyze Particles” plugin of the ImageJ software. Colocalization analysis was performed using the “Coloc2” plugin embedded in the ImageJ software. Analysis was performed on preselected whole cells. Each image was split into its respective *red*, *green*, and *blue* channels, and thresholding and mask conversion were performed as described previously. Mander's coefficients, M1 (representing the fraction of *red signal* [GABA_A_Rs] overlapping with *green signal* [GFP–gephyrin]) and M2 (representing the fraction of *green signal* overlapping with *red signal*) were calculated to determine the degree of overlap between the corresponding channels. Fluorescence intensity scans were obtained by drawing a straight line across GFP–gephyrin aggregates and microclusters of a cell in the green channel and measuring the intensity profile along the selected line in the green and red channel (GABA_A_Rs) using the “Plot Profile” plugin embedded in ImageJ.

### Transfections and immunocytochemistry of cultured hippocampal neurons

Cultures of hippocampal neurons were prepared from E18 CB KO mice and their WT littermates, as previously described (85). Neurons were transfected at DIV 4 using the CalPhos mammalian transfection kit (Clontech). Immunocytochemistry was performed as previously described (36, 61, 86). Multichannel image stacks of fluorescently stained neurons were acquired on an inverse Leica DMIRE2 microscope connected to a Leica TCS SP2 AOBS confocal laser scanning setup (Leica Microsystems) with constant settings, with a 63× oil-immersion objective and a zoom factor of 2. The image stacks were processed identically using the ImageJ software package (https://imagej.nih.gov/ij/). Briefly, “lei” files of the stacks were imported as Bio-Formats. Stacks of the different neurons were opened individually, autoscaled, and viewed with “Hyperstack.” The maximal intensity single channels of each image were obtained by using the “Z-Project” plugin embedded in the ImageJ software. Upon standardized thresholding and mask conversion, 40 μm dendritic segments were selected, and particles ≥0.05 μm^2^ as well as particle sizes were automatically analyzed by using the “Analyze Particles” plugin of the ImageJ software. Mander's coefficients, M1 (representing the fraction of *red signal* [mScarlet-α3^WT^] overlapping with *green signal* [endogenous gephyrin]) and M2 (representing the fraction of *green signal* overlapping with *red signal*), were calculated to determine the degree of overlap between the corresponding regions of detected signals at default settings. Region(s) of interest were defined around dendritic segments in the red channels (mScarlet-α3^WT^).

### Electrophysiology on cultured hippocampal neurons

Cultures of hippocampal neurons were prepared from E18 CB KO mice and their WT littermates, as previously described (85). Neurons were transfected at DIV 4 using the CalPhos mammalian transfection kit (Clontech) and grown for 14 to 16 DIV before electrophysiological analyses. Neurons were whole cell voltage clamped at −70 mV using a Multiclamp 700 B (Axon Instruments, Molecular Devices)/EPSC10 (HEKA electronics) amplifiers under the control of the pClamp software (Axon Instruments, Molecular Devices)/Patchmaster2 (HEKA electronics). Pipette resistances ranged between 3 and 4 MΩ, and only neurons with series resistances ≤12 MΩ were used for the analysis. Recordings of mIPSC were performed in the presence of 300 nM tetrodotoxin (Tocris Bioscience) and 10 μM 2,3-dihydroxy-6-nitro-7-sulphamoyl-benzo(F)quinoxaline (Tocris Bioscience) at holding potential −70 mV (87).

Cultured neurons were constantly supplied with fresh extracellular bathing solution (140 mM NaCl, 4 mM CaCl_2_, 4 mM KCl, 10 mM Hepes, 24 mM MgCl_2_, 10 mM glucose, adjusted to pH 7.3, and ~310 msOsmol/l) *via* a perfusion system. Neurotransmitter agonists (100 μM glutamate or 10 μM GABA) were applied using a fast flow application system. The intracellular solution contained 136 mM KCl, 17.8 mM Hepes, 15 mM phosphocreatine, 1 mM EGTA, 0.6 mM MgCl_2_, 0.3 mM Na-GTP, 4 mM Mg-ATP, and 5 U/ml creatine phosphokinase, adjusted to pH 7.4 (~320 mOsmol/l). All experiments were carried out at room temperature.

All electrophysiology data were analyzed using Axograph (version 1.5.4) software (Axograph Scientific).

### Immunohistochemistry

Immunolabeling of brain slices of CB KO mice and WT littermates was performed as previously described (64). Eight-week-old mice were deeply anesthetized and decapitated. The brains were immediately removed, cut sagitally in two equal halves, and frozen on dry ice. Sagittal cryostat sections (14 μm) were fixed with 4% (w/v) paraformaldehyde in PBS (pH 7.4) for 10 min at 4 °C, washed twice for 2 min in PBS and once with sodium citrate buffer (10 mM sodium citrate, 0.05% [v/v] Tween-20, pH 8.0). Subsequently, the sections were immersed in a preheated staining dish containing sodium citrate buffer and incubated for 30 min at 95 °C. After allowing the slides to cool down at room temperature for 20 min, sections were rinsed twice for 2 min in PBS, permeabilized with 0.3% (w/v) Triton X-100, 4% (v/v) goat serum in PBS, blocked for 3 h with 10% (v/v) goat serum in PBS, and incubated overnight at 4 °C with primary antibodies, and for 1 h with secondary antibodies at appropriate dilutions in PBS/10% (v/v) goat serum. Finally, the sections were incubated for 5 min in 2 nM 4′,6-diamidino-2-phenylindole in PBS, washed four times for 2 min in PBS, and mounted with Aqua Poly/Mount (Polysciences). Multichannel image stacks of fluorescently stained brain slices were acquired on an inverse Leica DMIRE2 microscope connected to a Leica TCS SP2 AOBS confocal laser scanning setup (Leica Microsystems) with constant settings, with a 63× oil-immersion objective and a zoom factor of 4. The image stacks were analyzed with the Imaris 9.6 software package. The fluorescently tagged protein puncta were modeled in 3D with ellipsoidal objects (*x* and *y* = 350 nm; *z* = 500 nm), whose creation parameters were kept constant during analysis of WT and KO slices. Briefly, parameters such as initial seed-point dimensions (*x* and *y* = 100 nm), quality (270 arbitrary units), and region threshold (20 arbitrary units) were kept constant. Quality of the spots (signal) describes the intensity of the channel Gaussian filtered by three-fourth of spot radius. Statistical parameters of the resulting spots (puncta) were extracted, including total spot number, diameter (minor axis length), spot volume, and fluorescence intensity. The spot density (spots per 100 μm^3^) was calculated on the basis of the total number of spots and the total volume of the image. Spots originating from different fluorescent stains were analyzed by the colocalized spots feature of Imaris (distance threshold = 1). The colocalizing spots were normalized to the total spot number to derive the percent of colocalizing spots.

### Statistics

Experimental data were evaluated by investigators blind to experimental conditions. Statistical significance was tested by using an unpaired two-tailed Student's *t* test. The required sample sizes (n) were estimated based on previous experiences with similar experiments (26, 36, 64). Values are represented as means ± SD. Asterisks indicate significant differences (∗*p* < 0.05; ∗∗*p* < 0.01; and ∗∗∗*p* < 0.001); ns indicates no significant difference. The statistics were evaluated using the GraphPad Prism software (GraphPad Software). Error estimates for ratios given in Figure 7, *B* and *C* were obtained by bootstrap analysis using 10,000 bootstrap resamples. Statistical significance of the shift in ratios observed between CB WT and CB KO cultures was assessed by permutation tests using 10,000 random permutations.

## Data availability

All data are contained within the article.

## Conflict of interest

The authors declare that they have no conflicts of interest with the contents of this article.
